# Psychometric properties of the Patient Assessment of Chronic Illness Care measure (PACIC-5A) among patients with obesity

**DOI:** 10.1186/s12913-019-3871-1

**Published:** 2019-01-23

**Authors:** Maria Schwenke, Franziska D. Welzel, Claudia Luck-Sikorski, Alexander Pabst, Anette Kersting, Matthias Blüher, Hans-Helmut König, Steffi G. Riedel-Heller, Janine Stein

**Affiliations:** 1 Institute of Social Medicine, Occupational Health and Public Health, Medical Faculty, University of Leipzig, Leipzig, Germany; 2 Integrated Research and Treatment Centre (IFB) AdiposityDiseases, University of Leipzig, Leipzig, Germany; 3 SRH University of Applied Sciences, Gera, Germany; 4 Department of Psychosomatic Medicine and Psychotherapy, University of Leipzig, Leipzig, Germany; 5 Department of Health Economics and Health Services Research, University Medical Center Hamburg-Eppendorf, Hamburg, Germany; 6 Institute of General Medicine, University of Leipzig, Leipzig, Germany

**Keywords:** Obesity, Primary care, Validity, Reliability, 5A counseling, PACIC

## Abstract

**Background:**

The Patient Assessment of Chronic Illness Care (PACIC-5A) was developed to assess the satisfaction with patient-provider interaction based on the Chronic Care Model. The additional 5A approach (assess, advise, agree, assist, arrange) allows to score behavioral counseling. The aim of the study was to assess the psychometric properties of the German adaptation of the PACIC-5A questionnaire in a sample of general practitioners (GP) patients with obesity.

**Methods:**

Analyses were based on data from the study “Five A’s counseling in weight management of obese patients in primary care: a cluster randomized controlled trial (INTERACT)”. Data were collected via standardized questionnaires containing the 26-item version of the PACIC-5A questionnaire. A total of 117 patients with obesity were included in the analyses. Statistical procedures comprised descriptive analyses, the calculation of Cronbach’s alpha, test-retest analyses and factor analyses in order to assess the psychometric properties including reliability and validity of the PACIC-5A.

**Results:**

The patient’s mean age was 43.4 years and the sample was mostly female (59%). Middle educational level was found for the majority (78%) and the mean Body Mass Index was 38.9 kg/m^2^. Descriptive analyses revealed a mean PACIC score of 2.33 and 5A sum score of 2.29. Notable floor effects were found. PACIC-5A showed high level of internal consistency (Cronbach’s alphas > 0.9) and exploratory factor analyses resulted in a unidimensional structure.

**Conclusion:**

The results of this study provide evidence regarding the psychometric properties of the German version of the PACIC-5A used in a sample of GP patients with obesity and make an important contribution to the reliable and valid assessment of the patient-GP interaction with regard to obesity counseling in primary care.

**Electronic supplementary material:**

The online version of this article (10.1186/s12913-019-3871-1) contains supplementary material, which is available to authorized users.

## Background

An increase in prevalence of chronic diseases is observed worldwide. According to the World Health Organization (WHO), obesity is one chronic non-communicable disease whose worldwide prevalence nearly tripled over the last 40 years [1]. In European regions, 23% of women and 20% of men are obese [2]. While the management of chronic diseases is mainly based on primary health care, general practitioners (GPs) treat patients with obesity on a regular basis and are responsible for both initial and long-term care. Improving the quality of the patient-GP interaction regarding obesity management is a key element for successful treatment of patients with obesity in general practice.

The Chronic Care Model (CCM) was developed for supporting and improving patient-centered primary care [3]. This multidimensional framework is based on six key dimensions: organization of health care, community resources, self-management-support, delivery system design, decision support and clinical information systems [3, 4]. On this basis, Glasgow et al. developed the 20-item self-administered Patient Assessment of Chronic Illness Care (PACIC) to assess the satisfaction with patient-provider interaction from the patient’s perspective [5]. The authors predefined five subscales based on the CCM and evaluated PACICs’ reliability and validity among patients with at least one chronic illness. Reliability was satisfying and results of the confirmatory factor analysis (CFA) confirmed the predefined structure with moderate goodness of fit [5]. In recent years, the instrument has been translated into several languages, including German, Spanish, Dutch, Danish and French and several studies investigated the psychometric properties among patients with different chronic diseases such as diabetes, arthritis, hypertension and chronic obstructive pulmonary disease [6–10]. Overall, PACIC investigations suggested acceptable reliability but structure validity through CFA and exploratory factor analysis (EFA) showed conflicting results. While few studies supported the five-factor structure [6, 7, 10], others could not confirm it [9, 11–15].

The patient-centered “5As” (assess, advise, agree, assist, arrange) model represents an evidence-based approach of behavioral counseling [16, 17]. Congruent with the CCM, the 5As are used to improve self-management support [18].The approach was originally developed for smoking cessation counseling. Nowadays, it has been adapted and used for different dependency-related conditions and chronic diseases including obesity [19]. The PACIC-5A represents an advanced version of the PACIC and was extended by six items to assess the 5A model according to the recommendations of the US Preventive Services Task Force forming five subscales reflecting CCM as well as further five subscales reflecting the 5As and total scores [18]. Glasgow et al. evaluated the PACIC-5A questionnaire among diabetic patients. Internal consistency showed good results for the 5A summary score (α > 0.9) and adequate variability and distribution of the scales were shown. However, investigations of construct validity of the PACIC-5A are pending [18]. Recently, a study in Germany examined psychometric properties of PACIC-5A including construct validity through EFA [10]. While the structure as proposed by Glasgow et al. was confirmed, no detailed results of the factor analysis were shown in this study [10]. Other previous studies used the PACIC-5A to evaluate chronic care, especially for diabetic and asthmatic patients [20–23]. However, little is known about the underlying structure of the instrument so far.

Taken together, previous studies showed inconsistent results with regard to the psychometric properties of PACIC and only two studies evaluated PACIC-5A with gaps in analyses of the construct validity. Further, PACIC and PACIC-5A was mostly used and validated in samples of diabetic patients. So far, little is known about the psychometric quality of the PACIC-5A used in patients with obesity. Thus, the aim of the current study was to conduct a psychometric analysis of the German adaptation of the PACIC-5A scale in a sample of patients with obesity in primary care.

## Methods

### Study design and sample

This methodological study used data from the study “Five A’s counseling in weight management of patients with obesity in primary care: A cluster-randomized controlled trial (INTERACT)” [24]. The INTERACT study is a cluster-randomized controlled trial aiming at the implementation and evaluation of the internet-based learning program “5A Adipositas Management” (The 5A’s of obesity management) in order to improve weight counseling within the German primary health care setting. Participants were assessed at baseline assessment (BL) and two follow-up (FU) assessments after 6 and 12 months. In addition, data from general practitioners (GP) were collected at baseline and after 12 months. Patients were recruited via GPs within their practices during consultation following specific inclusion criteria: (1) Body Mass Index (BMI) equal or greater than 30 kg/m^2^, (2) age between 18 and 60 years and (3) German as native language. Patients were excluded from participation if acute physical or mental illnesses required priority management and made study participation impossible according to the attending GP. More detailed information of the INTERACT study has been reported elsewhere [24].

**Fig. 1:**
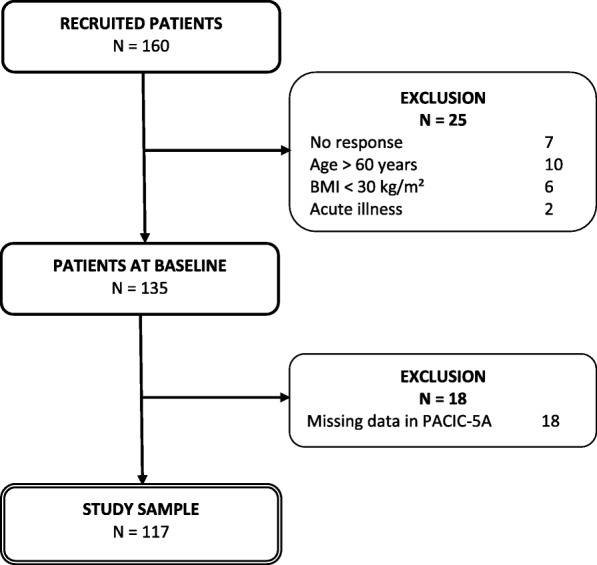
Flowchart of the sample selection

For the project, 160 subjects from 39 general practices in Central Germany were recruited. 25 patients were excluded because of BMI < 30 kg/m^2^ (*n* = 6), Age > 60 years (*n* = 10), no baseline response (*n* = 7) and two patients had an acute illness that needed priority management, thus the sample at BL included 135 patients. In the current study, further 18 (13.3%) patients were excluded from the sample because of missing values in the PACIC-5A questionnaire. All analyses of this study were based on a sample of 117 patients at BL. The sample selection process is shown in Fig. 1.

### Procedure and instruments

Following the given criteria for inclusion and exclusion, the patients were selected by GPs and asked to take part in the study. Written informed consent and basic patient information was obtained from participants and sent back to the research staff. After receiving the documents, standardized self-rating questionnaires were sent to patients by postal mail. The patients were asked to complete all questionnaires at home and sent it back to the study center. The collection of data covered sociodemographic variables including age, gender, weight/height, and education (low, middle, high) according to the new CASMIN educational classification [25]. The patient-physician interaction regarding obesity management was assessed by using the German adaptation of the Patient Assessment of Chronic Illness Care (PACIC-5A), which is an instrument to estimate the quality of chronic illness care according to the CCM [18]. Additionally, the PACIC-5A can be used to assess the model for behavior counseling called “5A”. It includes 26 items that can be scored on a 5-point Likert scale ranging from “1 = Almost never” to “5 = Almost always”. The first 20 items of the PACIC-5A can be aggregated into five subscales based on the key components of the CCM: Patient Activation (Items 1–3), Delivery System Design/Decision Support (Items 4–6), Goal Setting (Items 7–11), Problem-solving/Contextual Counseling (Items 12–15), Follow-up/Coordination (Items 16–20) and an overall PACIC score. By including the further six items, the instrument can also be grouped into the 5A summary score as well as five subscales that cover elements of the 5A approach: Assess (Item 1, 11, 15, 20, 21), Advise (Item 4, 6, 9, 19, 24), Agree (Item 2, 3, 7, 8, 25), Assist (Item 10, 12, 13, 14, 26), Arrange (16, 17, 18, 22, 23). In addition, for each patient specific data including comorbidities and height/weight were collected via standardized GP questionnaires. The study was approved by the ethic committee of the University of Leipzig and written informed consent was collected from all participants.

### Statistical analyses

The data analyses were conducted using SPSS Statistics 24.0 (Statistical Package for Social Science Inc., IBM®, Chicago, IL). The overall PACIC score was calculated as the mean value of the first 20 items; the 5A summary score was the mean value of items 1–4 and 6–26. Each subscale was scored by averaging them across the corresponding items as described previously [18]. Scores can range from 1 to 5 and higher scores indicate a higher quality of chronic illness care from patient’s perspective. Statistical procedures comprised descriptive analyses of patient characteristics and the PACIC-5A items, subscales and total scores including means, standard deviations or absolute and relative frequencies with percentages, as appropriate. Floor and ceiling effects of the PACIC-5A were analyzed via the response rates of the lowest or highest possible category. Associations between the overall PACIC and 5A summary scores and patient characteristics were analyzed via Pearson product-moment correlation and Spearman’s rank correlation, as appropriate*.* In order to assess aspects of reliability, test-retest reliability by using Intraclass Correlation Coefficient (ICC) and Cronbach’s alpha were calculated for the total scores and the subscales. Acceptable values for Cronbach’s alpha range from 0.70 to 0.95 [26]. Exploratory factor analysis with principal component analysis was conducted in order to analyze the structure of the questionnaire and the construct validity. Prior to factor analysis, tests of multicollinearity (Bartlett test of sphericity with *p*-value < 0.05) were run and sampling adequacy was calculated (Kaiser-Meyer-Olkin-criterion (KMO) ≥ 0.50) in order to examine criteria of feasibility. The number of factors was determined by using eigenvalue > 1 (Kaiser criterion), scree plot and parallel analysis (PA) following O’Connor’s SPSS syntax [27]. We used oblique (promax) rotation where more than one factor was identified. Additionally we conducted a confirmatory factor analyses (CFA) to test the predefined factor structure with maximum likelihood estimation method. The model fit was tested using Comparative Fit Index (CFI; acceptable fit ≥0.95), Root Mean Square Error of Approximation (RMSEA; acceptable fit ≤0.06) and Standardized Root Mean Residual (SRMR, acceptable fit ≤0.08) [28]. The CFA were performed using Stata 15.1 MP (Stata Corp LP, College Station, TX). Statistical significance was assumed at *p*-value ≤0.05 for all computations.

## Results

In Table 1, characteristics of the patient sample are displayed. Mean age of patients was 43.4 (SD =10.7) years ranging from 20 to 60 years. The majority of the sample was female (59%) and had middle educational level (66.7%). The mean BMI was 38.9 ± 6.0 kg/m^2^, whereas 27% of the patients were classified as obese class I (BMI 30.0–34.9 kg/m^2^), 39% as obese class II (BMI 35.0–39.9 kg/m^2^) and 34% as obese class III (BMI >  40 kg/m^2^) according to the WHO classification [29]. The average number of comorbidities according to the GPs was 4.6 ± 3.2 in addition to obesity.

**Table 1:** Patient characteristics (*N* = 117)

Characteristics	Values
Age in years, Mean (SD)	43.4 (10.7) Range 20–60
Sex, n (%)	
Male	48 (41.0)
Female	69 (59.0)
Education^a^, n (%)	
Low	28 (23.9)
Middle	78 (66.7)
High	11 (9.4)
BMI in kg/m^2^, Mean (SD)	38.9 (6.0) Range 30.4–56.6
BMI classification^b^, n (%)	
Obesity class I (30–35.9 kg/m^2^)	32 (27.0)
Obesity class II (35–39.9 kg/m^2^)	45 (39.0)
Obesity class III (> 40 kg/m^2^)	40 (34.0)
Number of comorbidities, Mean (SD)	4.6 (3.2) Range 0–14

*BMI* Body Mass Index, *SD* standard deviation, ^a^education classification according CASMIN classification, ^b^BMI classification according World Health Organization

To assess associations between the patient characteristics and the overall PACIC and 5A summary scores correlations were calculated (Table 2). In almost all cases the correlation coefficients were close to zero. Weak negative correlation was observed between the scores and BMI (r(PACIC) = − 0.140, r(5A) = − 0.138). The number of comorbidities (r(PACIC) = 0.136, r(5A) = 0.128) were slightly positive correlated with the scores, but none of these correlations were significant (Table 2).

**Table 2:** Correlations between overall PACIC score and 5A summary score with patient characteristics (*N* = 117)

	Overall PACIC score		5A summary score	
	Rho^a^	*p*-value	Rho^a^	*p*-value
Age	−0.089	0.338	−0.093	0.317
Sex	0.078	0.400	0.084	0.370
Education	−0.072	0.443	−0.064	0.493
BMI	−0.140	0.133	−0.138	0.136
BMI classification	−0.095	0.311	−0.094	0.311
Number of comorbidities	0.136	0.145	0.128	0.169

^a^Pearsons product-moment correlation or Spearman rank correlation as appropriate

Table 3 shows the descriptive statistics and the results of the item and scale analyses of the PACIC-5A scores as proposed by Glasgow et al. [18]. Descriptive analyses revealed a mean overall PACIC score of 2.33 ± 0.8 with individual PACIC items ranging from 1.42 ± 0.9 (Item 9) to 3.46 ± 1.3 (Item 5). The predefined PACIC subscales varied from 2.08 ± 0.9 (Follow-up/coordination) to 2.77 ± 1.0 (Delivery system/Decision Support).

**Table 3:** Descriptive items and scale characteristics (*N* = 117)

Scales	Mean (SD)	Floor effects n (%)	Ceiling effects n (%)	Item-scale-correlation^a^	Item-total-correlation^b^	α	Test- retest ICC (*n* = 56)
Overall PACIC Score	2.33 (0.8)	6 (5.1)	–			0.93	0.57
Patient Activation	2.70 (1.1)	10 (8.5)	3 (2.6)			0.72	0.46
Item 1	2.88 (1.4)	27 (23.1)	19 (16.2)	0.63	0.73		
Item 2	2.50 (1.4)	40 (34.2)	12 (10.3)	0.66	0.66		
Item 3	2.72 (1.5)	37 (31.6)	18 (15.4)	0.37	0.52		
Delivery System/ Decision Support	2.77 (1.0)	13 (11.1)	2 (1.7)			0.71	0.30
Item 4	1.82 (1.2)	70 (59.8)	4 (3.4)	0.40	0.49		
Item 5	3.46 (1.3)	17 (14.5)	28 (23.9)	0.60	0.64		
Item 6	3.03 (1.2)	20 (17.1)	14 (12.0)	0.59	0.59		
Goal Setting/ Tailoring	2.11 (0.9)	22 (18.8)	–			0.75	0.46
Item 7	2.94 (1.4)	28 (23.9)	20 (17.1)	0.69	0.74		
Item 8	2.69 (1.4)	35 (29.9)	15 (12.8)	0.66	0.71		
Item 9	1.42 (0.9)	89 (76.1)	2 (1.7)	0.40	0.50		
Item 10	1.79 (1.2)	72 (61.5)	5 (4.3)	0.40	0.45		
Item 11	1.71 (1.1)	75 (64.1)	3 (2.6)	0.43	0.45		
Problem Solving/ Contextual	2.28 (1.2)	28 (23.9)	2 (1.7)			0.89	0.59
Item 12	2.67 (1.5)	40 (34.2)	15 (12.8)	0.72	0.79		
Item 13	1.82 (1.1)	66 (56.4)	3 (2.6)	0.71	0.71		
Item 14	2.14 (1.3)	55 (47.0)	7 (6.0)	0.83	0.78		
Item 15	2.50 (1.4)	42 (35.9)	11 (9.4)	0.77	0.76		
Follow-up/ Coordination	2.08 (0.9)	22 (18.8)	–			0.70	0.60
Item 16	1.57 (1.1)	88 (75.2)	6 (5.1)	0.13	0.28		
Item 17	2.27 (1.3)	47 (40.2)	10 (8.5)	0.46	0.50		
Item 18	2.09 (1.4)	63 (53.8)	12 (10.3)	0.53	0.53		
Item 19	1.92 (1.3)	69 (59.0)	10 (8.5)	0.66	0.51		
Item 20	2.55 (1.6)	50 (42.7)	19 (16.2)	0.52	0.54		
5A Summary Score	2.29 (0.9)	6 (5.1)	–			0.94	0.63
Assess	2.45 (1.0)	14 (12.0)	–			0.80	0.57
Item 1	2.88 (1.4)	27 (23.1)	19 (16.2)	0.61	0.72		
Item 11	1.71 (1.1)	75 (64.1)	3 (2.6)	0.40	0.47		
Item 15	2.50 (1.4)	42 (35.9)	11 (9.4)	0.69	0.78		
Item 20	2.55 (1.6)	50 (42.7)	19 (16.2)	0.53	0.55		
Item 21	2.62 (1.4)	36 (30.8)	13 (11.1)	0.69	0.78		
Advise	2.29 (0.8)	10 (8.5)	–			0.70	0.54
Item 4	1.82 (1.2)	70 (59.8)	4 (3.4)	0.40	0.46		
Item 6	3.03 (1.2)	20 (17.1)	14 (12.0)	0.51	0.58		
Item 9	1.42 (0.9)	89 (76.1)	2 (1.7)	0.46	0.50		
Item 19	1.92 (1.3)	69 (59.0)	10 (8.5)	0.41	0.52		
Item 24	3.28 (1.4)	20 (17.1)	31 (26.5)	0.51	0.74		
Agree	2.67 (1.1)	9 (7.7)	1 (0.9)			0.84	0.55
Item 2	2.50 (1.4)	40 (34.2)	12 (10.3)	0.63	0.67		
Item 3	2.72 (1.5)	37 (31.6)	18 (15.4)	0.44	0.53		
Item 7	2.94 (1.4)	28 (23.9)	20 (17.1)	0.73	0.75		
Item 8	2.69 (1.4)	35 (29.9)	15 (12.8)	0.71	0.71		
Item 25	2.50 (1.4)	42 (35.9)	11 (9.4)	0.73	0.76		
Assist	1.99 (0.9)	31 (26.5)	–			0.81	0.50
Item 10	1.79 (1.2)	72 (61.5)	5 (4.3)	0.39	0.45		
Item 12	2.67 (1.5)	40 (34.2)	15 (12.8)	0.72	0.82		
Item 13	1.82 (1.1)	66 (56.4)	3 (2.6)	0.73	0.72		
Item 14	2.14 (1.3)	55 (47.0)	7 (6.0)	0.74	0.80		
Item 26	1.51 (1.1)	90 (76.9)	7 (6.0)	0.45	0.47		
Arrange	2.02 (0.9)	25 (21.4)	–			0.68	0.69
Item 16	1.57 (1.1)	88 (75.2)	6 (5.1)	0.23	0.29		
Item 17	2.27 (1.3)	47 (40.2)	10 (8.5)	0.47	0.50		
Item 18	2.09 (1.4)	63 (53.8)	12 (10.3)	0.35	0.53		
Item 22	2.29 (1.4)	53 (45.3)	11 (9.4)	0.51	0.64		
Item 23	1.88 (1.3)	68 (58.1)	7 (6.0)	0.61	0.67		

*SD* standard deviation, *PACIC* Patient Assessment of Chronic Illness Care, *α* Chronbach’s alpha, *ICC* Intraclass Correlation Coefficient, ^a^correlation between item and referring scale, ^b^correlation between item and overall PACIC score or 5A summary score

The mean 5A summary score was 2.29 ± 0.9 with individual 5A items ranging from 1.42 ± 0.9 (Item 9) to 3.28 ± 1.4 (Item 24). Grouped into the 5A subscales values between 1.99 ± 0.9 (Assist) to 2.67 ± 1.1 (Agree) were observed.

**Fig. 2:**
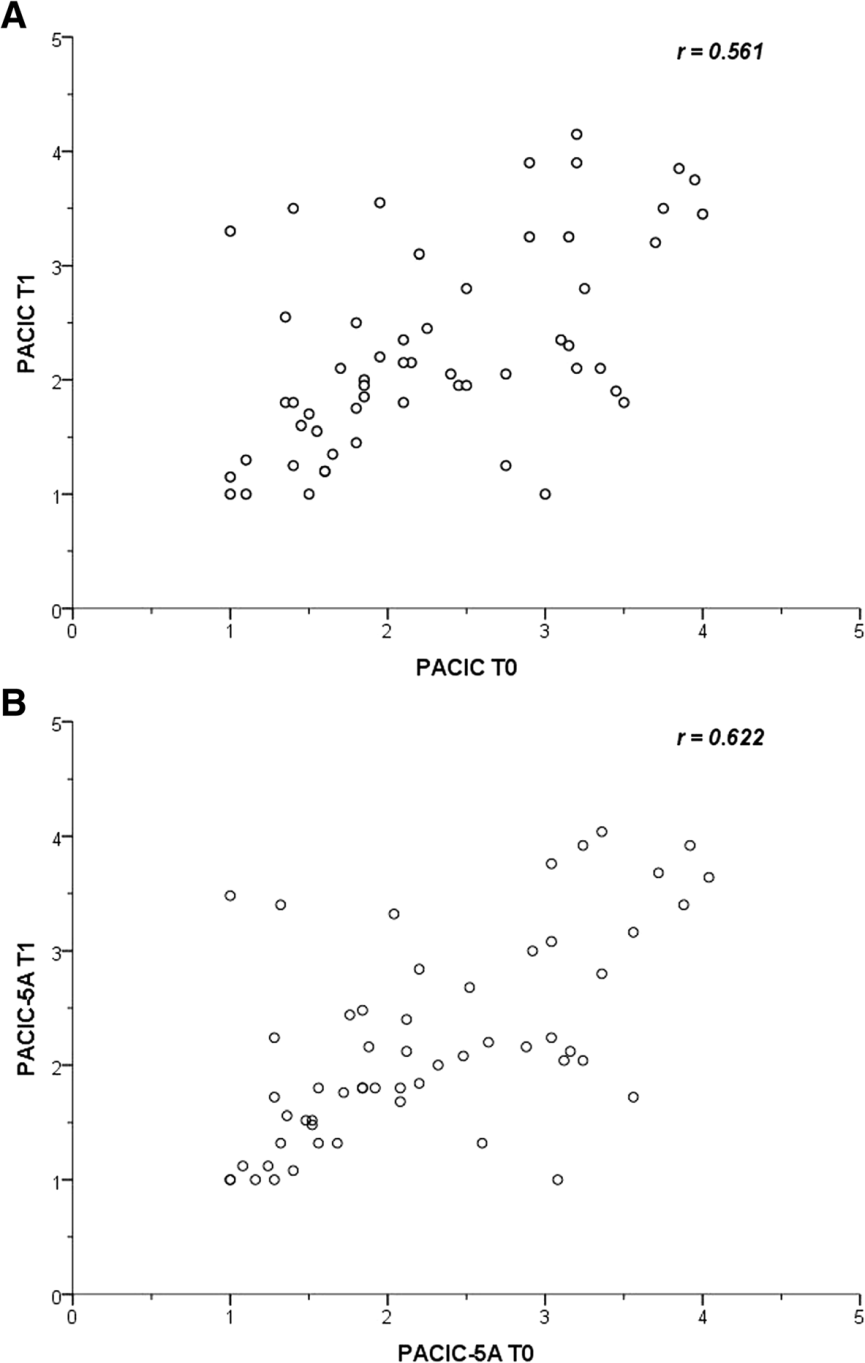
Test-retest scatterplot of (**a**) PACIC and (**b**) 5A with T0 baseline measurement and T1 6-month measurement

Floor effects for overall PACIC score and 5A summary score were 5.1% in each case and no ceiling effects were detected. For individual items the percentage of persons who used the lowest answering category (“almost never”) ranged from 14.5 to 76.9% and was in 23 of 26 items above 20%. The percentage of persons who used the highest answering category (“almost always”) ranged from 1.7 to 26.5% at item level and was in two items above 20%. Internal consistency in terms of Cronbach’s alpha achieved 0.93 for the overall PACIC score and 0.94 for the 5A summary score. For the individual subscales Cronbach’s alpha ranged from 0.68–0.89 and reached mostly the threshold of 0.70. An exception was the 5A Arrange subscale with an alpha just below the threshold (0.68). The correlation between individual items and the referring scale (item-scale correlation) varied from *r* = 0.13 to 0.83 and was in items of two PACIC subscales and two 5A subscales under the rule-of-thumb minimum value of 0.4 (Patient activation, Follow-up/ Coordination, Assist and Arrange). The correlation between individual items and the total scores (item-total correlation) was in almost all items over 0.4. Only item 16 had values below 0.3 for overall PACIC score and 5A summary score. Cronbach’s alpha if item 16 deleted changed marginally from 0.925 to 0.927 for overall PACIC score and from 0.944 to 0.946 for 5A summary score. To assess test-retest reliability, ICCs were measured in a sample of 56 patients six month after baseline survey. ICC for overall PACIC score was 0.57 and ranged from 0.30 to 0.60 for the subscales. Test-retest reliability for the 5A scales reached higher values with 0.63 for 5A summary score and 0.50 to 0.69 for the subscales. In addition, we presented the values of the two measurement times of the total scores in a scatter plot and calculated pearson’s correlation (Fig. 2). The values of pearson’s correlation are comparable to the ICCs (r(PACIC) = 0.56; r(5A) = 0.62).

**Fig. 3:**
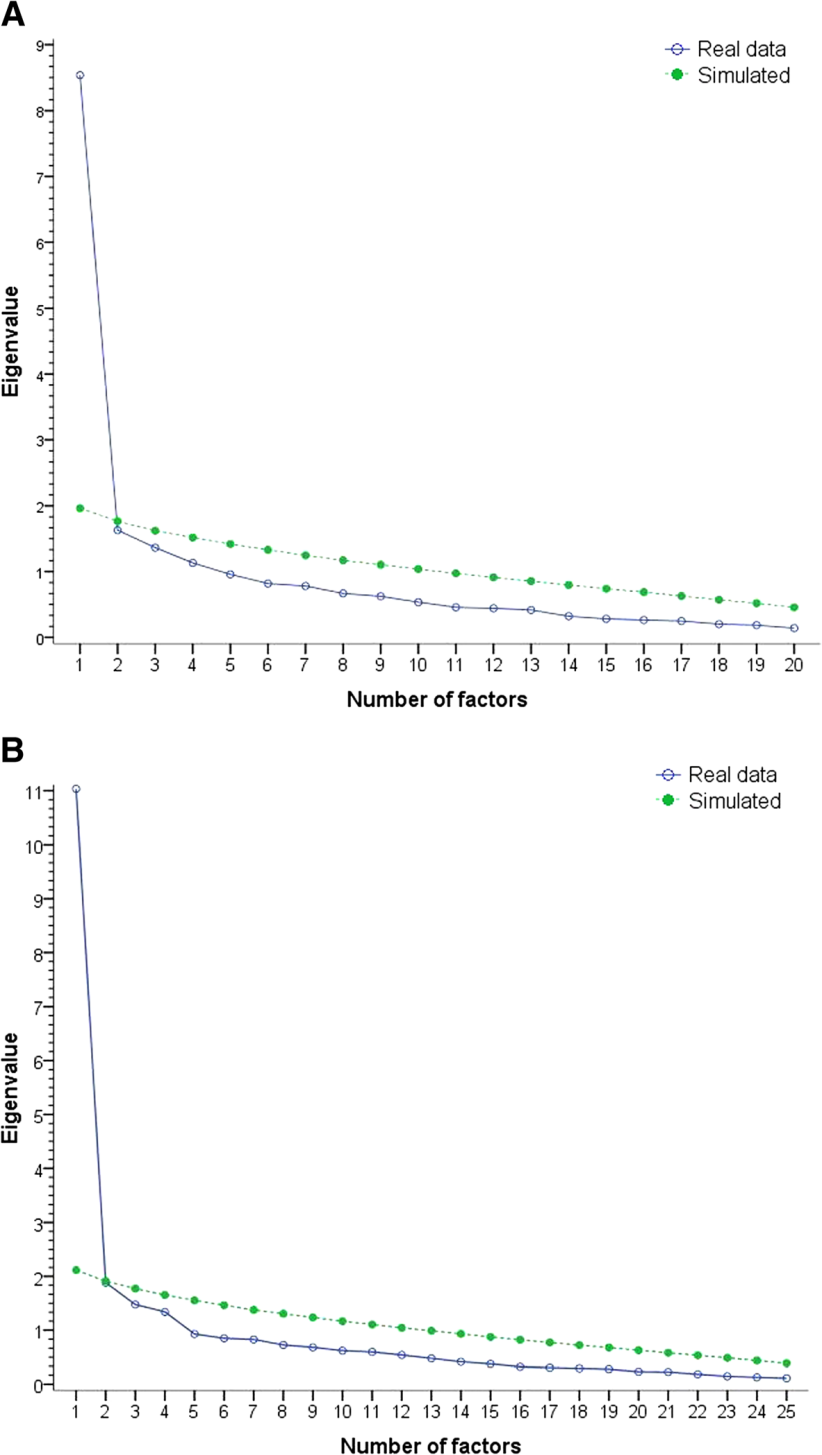
Parallel analyses of (**a**) PACIC (20 items) and (**b**) 5A (25 items)

The criteria of feasibility of factor analysis showed good fit (Bartlett tests of sphericity *p* = 0.000, KMO (PACIC) = 0.888, KMO (5A) = 0.895). The Kaiser-Meyer-Olkin measure of each item revealed values above 0.7, which is shown in Table 4. Factor analysis with eigenvalue criterion identified four factors. Factor loadings after promax rotation for the four factors are shown in the Additional file 1: Table S1 and S2. However, scree-plot and parallel analysis showed one factor structures in each case (Fig. 3). Furthermore a content analysis of the four factors did not provide a meaningful structure of the underlying concepts, so that the one factor structure convinced sufficiently. The first factor was most prominent and had an eigenvalue of 11 explaining 44% of the variation of the 5A structure (PACIC: eigenvalue 8.5 and 43% explained variation). In Table 4, the results of the factor analysis for one factor structure of the 20 PACIC items and the 25 5A items are shown. Factor loadings were above 0.5 in 90% of the PACIC items and 88% of the 5A items, supporting the one factor structure. Only item 16 had factor loadings < 0.4 in each case.

**Table 4:** Factor loadings and measure of sampling adequacy of PACIC and 5A for one-factor-structure

Items		PACIC		5A	
		Factor loadings	KMO	Factor loadings	KMO
1	Asked for my ideas when we made a treatment plan	0.778	0.898	0.759	0.914
2	Given choices about treatment to think about	0.719	0.896	0.709	0.914
3	Asked to talk about any problems with my medicines or their effects.	0.562	0.905	0.564	0.923
4	Given a written list of things I should do to improve my health.	0.530	0.780	0.493	0.772
5	Satisfied that my care was well organized.	0.686	0.935		
6	Shown how what I did to take care of my illness influenced my condition.	0.642	0.913	0.618	0.932
7	Asked to talk about my goals in caring for my illness.	0.792	0.936	0.785	0.940
8	Helped to set specific goals to improve my eating or exercise.	0.759	0.931	0.753	0.912
9	Given a copy of my treatment plan.	0.550	0.834	0.536	0.838
10	Encouraged to go to a specific group or class to help me cope with my chronic illness.	0.501	0.886	0.489	0.814
11	Asked questions, either directly or on a survey, about my health habits.	0.492	0.873	0.503	0.823
12	Sure that my doctor or nurse thought about my values and my traditions when they recommended treatments to me.	0.829	0.966	0.847	0.954
13	Helped to make a treatment plan that I could do in my daily life.	0.757	0.902	0.749	0.900
14	Helped to plan ahead so I could take care of my illness even in hard times.	0.826	0.895	0.829	0.898
15	Asked how my chronic illness affects my life.	0.804	0.927	0.811	0.932
16	Contacted after a visit to see how things were going.	0.319	0.714	0.322	0.734
17	Encouraged to attend programs in the community that could help me.	0.540	0.871	0.522	0.837
18	Referred to a dietitian, health educator, or counselor.	0.577	0.761	0.562	0.776
19	Told how my visits with other types of doctors, like the rheumatologist or orthopedic surgeon, helped my treatment.	0.545	0.790	0.549	0.803
20	Asked how my visits with other doctors were going.	0.571	0.857	0.580	0.896
21	Asked what I would like to discuss about my illness at that visit.			0.806	0.951
22	Asked how my work, family, or social situation related to taking care of my illness.			0.684	0.894
23	Helped to make plans for how to get support from my friends, family or community.			0.701	0.895
24	Told how important the things I do to take care of my illness (e.g. exercise) were for my health.			0.771	0.942
25	Set a goal together with my team for what I could do to manage my condition.			0.797	0.908
26	Given a book or monitoring log in which to record the progress I am making.			0.508	0.900

*KMO* Kaiser-Meyer-Olkin criterion, *PACIC* Patient Assessment of Chronic Illness Care

We tested the unidimensional structures as well as Glasgow’s five-factor structures via CFA. Results are shown in Table 5. Chi-squared-tests did not reach significance levels, which indicated that perfect model fit is missing. All models had values close to the cut-off value for SRMR (≤ 0.08), whereby the unidimensional models reached the threshold. The model fit was not acceptable measured by the fit indices RMSEA and CFI. In summary, fit indices of CFA could confirm neither the five-factor model nor the unidimensional model with adequate fit.

**Table 5:** Model fit of the Confirmatory Factor Analyses (N = 117)

	PACIC		5A	
	1-factor model	5-factor model	1-factor model	5-factor model
Χ^2^ (df)	402.01 (170)	322.78 (160)	632.07 (275)	606.82 (265)
*p*-value	< 0.001	< 0.001	< 0.001	< 0.001
CFI	0.805	0.863	0.792	0.801
RMSEA (90% CI)	0.108 (0.094–0.122)	0.093 (0.079–0.108)	0.105 (0.095–0.116)	0.105 (0.094–0.116)
SRMR	0.080	0.082	0.079	0.083

*df* degrees of freedom, *CFI* Comparative Fit Index, *RMSEA* Root Mean Square Error of Approximation, *CI* confidence interval, *SRMR* Standardized Root Mean Residual

## Discussion

The present study aimed at evaluating the psychometric properties for the German version of the PACIC-5A questionnaire among patients with obesity in primary care. To our knowledge, this was the first study that investigated reliability and validity of the instrument used in patients with obesity. As a main result, our study showed satisfactory evidence with regard to psychometric properties of the German language version of the PACIC-5A questionnaire used in the context of obesity management.

The mean PACIC scores of previous validation studies ranged from 2.4 to 3.2 [5, 10]. In comparison, our results showed a slightly lower value with 2.3 of possible 5 for overall PACIC score. The value is under the center point of the scale indicating tendencies that elements of the Chronic Care Model (CCM) are generally not met in primary care according to patient’s perspective. The result for the present 5A summary score was also 2.3 of possible 5. In contrast, Glasgow et al. revealed a comparably higher 5A summary score of 3.2 in a sample of diabetic patients and Rosemann et al. showed a value of 2.5 among patients with osteoarthritis [10, 18]. In turn, one recently published study that used PACIC-5A among asthmatic patients in Switzerland revealed lower values of 2.0 for overall PACIC score and 5A summary score at baseline [23]. The lower scores in the present study were accompanied by high floor effects. On the one hand, high floor effects may indicate a low sensitivity range of the instrument in the lower categories [30]. On the other hand, it could represent the reality with an absence of specific elements of the CCM.

Originally, Glasgow et al. hypothesized that PACIC should not be related to patients’ demographics but to disease characteristics [5]. Indeed, our study results confirmed the first part of Glasgow’s hypotheses because we found no significant associations between overall PACIC score or 5A summary score and patients characteristics including sex, age and education. Between the number of comorbidities and total scores we found weak positive correlation, but these results did not reach statistical significance in present study. Thus, the second part of Glasgow’s hypothesis could not be confirmed based on our results. Almost all previous studies that analyzed the association between overall PACIC score and number of chronic conditions [5, 6, 8, 10, 13, 18, 31] showed no significant or inconsistent associations. Only Glasgow et al. found weak significant correlations in one study but could not confirm these findings in another study [5, 18]. Further, some studies that used PACIC or PACIC-5A considered patients’ weight for descriptive statistics [21, 32–34] and two studies investigated the correlation between BMI and PACIC-5A or PACIC short form, but they did not find significant associations [35, 36]. Similar to these findings, we found weak negative correlations between the total scores and BMI, but the results were not significant.

Regarding internal consistency measured by Cronbach’s alpha, our results were similar to others and showed high reliability values for overall PACIC and 5A summary scores. In line with previous validation studies [5, 7, 37], Cronbach’s alpha for overall PACIC score was 0.93 in this study. For the 5A summary score we revealed a Cronbach’s alpha of 0.94 which was slightly lower compared to the findings of Glasgow et al. (0.97) but higher than the results of Rosemann et al. (0.83) [10, 18]. Internal consistency of the subscales also showed good reliability with values ranging from 0.7–0.9. However, the item-scale-correlations of a few items gave some reason for concern. For example, item 16 in the “Follow-up/Coordination” subscale as well as in the 5A subscale “Arrange” showed correlations of 0.13 and 0.24. Similar results are shown for the correlation between the total scores and the items. Overall, the item-total correlations were acceptable except for item 16. However, Cronbach’s alpha did not change notably if item deleted, thus we decided to keep the item in the scales. The item is already known to not fit well into the structure like mentioned in Glasgow’s validation study [5]. Indeed, it was considered as important item for follow-up scale and retained according to Glasgow et al. [5]. Based on these findings one may assume that some items do not sufficiently reflect the intended meaning of the subscale*.* Consequently, this should be taken into account when using the subscales and further studies are necessary. PACIC’s test-retest reliability over a six-month interval was moderate and comparable to results of Glasgow’s validation study (3-month test-retest reliability = 0.58) [5]. 5A scores demonstrated higher ICC values in our analyses and were slightly lower than in study of Rosemann et al. (2-weeks test-retest reliability = 0.88) [10]. Differences could be explained by variations in the retest intervals, whereby the three-month interval is more comparable to our interval.

We found 18 studies that investigated the structure of PACIC with different methods while most of them are in accordance with present results and could not confirm the predefined five-factor structure as proposed by Glasgow et al. [5–15, 31, 32, 35, 37–40]. Three of the 18 studies found a two-factor structure by using exploratory factor analysis EFA [9, 12, 13] and one study conducted an EFA with best fit for four-factor structure with a prominent first factor that included more than half of all items [15]. Furthermore, eight of the 18 studies suggested a one-factor structure and use of the overall PACIC score [11, 14, 31, 32, 35, 37–39]. Three of the eight studies used EFA partly with parallel analysis (PA), which was similar to our investigation [11, 32, 38]. One of the eight studies applied principal component analysis (PCA) but had a look only on the PACIC short form [39] and two studies used confirmatory factor analysis (CFA) and showed acceptable to good fit for PACIC and PACIC short form [35, 37]. Moreover, two of the eight studies tested different structure models via CFA including the predefined five-factor structure but none had acceptable model fit [14, 31], which is in line with present CFA results. Therefore, the authors recommended the calculation and use of the overall PACIC score. In contrast to our results and mentioned studies, Glasgow et al. developed and confirmed their predefined five-factor model by CFA with moderate fit [5]. Further five studies among the 18 studies suggested also a five-factor structure, whereby the results were not throughout uniform and not always equal to the originally developed structure. One study that used PCA confirmed only three of the five predefined subscales [7]. Another study used CFA and reported poor fit for four indices and good fit for two indices [8]. Two studies that used EFA confirmed Glasgow’s five-factor structure but did not provide detailed information about analysis strategy and results [6, 10]. Noël et al. used EFA and CFA to identify a five-factor structure but the distribution was different from the predefined suggestion [40]. In summary, many studies evaluated the structure of PACIC but there is conflicting evidence. Overall, most of the studies suggested a one- to two-factor structure which goes in hand with the present results for EFA. The statistical methods between the studies were quiet different and studies that conducted EFA used different methods to estimate the number of factors. We used EFA with PA as well as eigenvalue criterion and scree plot. Decisive was the PA because it is one of the most accurate methods [27, 41]. Contrary to the PA, the scree-plot is more subjective and the eigenvalue criterion often overestimates the number of factors, thus these criteria are assumed to be less exactly than PA [27, 41].

In contrast to the numerous studies on the PACIC structure, only one previous study investigated the structure of PACIC-5A. Although Glasgow et al. defined the 5A subscales according to the US Preventive Services Task Force and developed PACIC-5A, they did not evaluate the underlying structure. Only Rosemann et al. investigated and confirmed the structure of the 5A concept of PACIC-5A via EFA but little is known about the analytic and detailed results [10]. In our analysis, the five proposed subscales of the 5A construct could not be confirmed by confirmatory and exploratory factor analyses. Thus, more investigations are necessary to confirm the 5As in PACIC-5A among different patient samples.

In summary, we could not confirm Glasgow’s 5-factor structure for PACIC and 5A. Our exploratory factor analyses resulted in one-factor solutions according to PA and scree-plot. Furthermore, one fit index of conducted CFA reached the threshold for the one-factor structure. Thus, in line with previous studies [11, 14, 31, 32, 37] we recommend the use of the total scores to assess patient-providers interactions. However, it must be mentioned that other CFA indices showed poor fit.

It is important to note that the present study had some limitations. First, our study had a sample size of 117 persons which is rather in the lower range compared with other PACIC validation studies. It has to be mentioned that fit indices of CFA are vulnerable for small sample sizes and tend to over-reject models, thus Hu and Bentler recommended samples with more than 250 subjects [28]. Second, PACIC was measured via ordinal 5-point Likert scale though we used the scales like metric variables. Some PACIC validation studies criticized that the ordinal nature of the data is often not considered [11, 14], but a condition that a Likert scale could trait as “quasi-metric” is that there are at least five scale categories, what is given in the used PACIC-5A questionnaire [42, 43]. However, our analyses showed similar results like studies that took the ordinal structure into account [32, 35, 38]. Third, we were not able to analyze other aspects of reliability and validity such as criterion validity based on our data. However, no accepted gold standard instrument is available for comparison.

The reimbursement system of the health insurances in Germany covers illnesses which occur subsequently or are associated with obesity but not obesity as a single disease entity [44]. This aspect could be a further explanation for the low mean values of PACIC-5A in present study and calls for optimization of obesity care in Germany. In accordance, the WHO declared obesity as disease since 2000 [29] and in 2011 the European Parliament urged the uniform approval of obesity as chronic condition for adequate treatment and prevention [45].

## Conclusions

In conclusion, the results provide substantial evidence regarding the psychometric properties of the German version of the PACIC-5A as practicable instrument for the assessment of primary care structure and self-management support in obesity from patient’s perspective. Further studies should preferably use the overall scores. The subscales should be viewed with caution and may be useful for comparison in follow-up examinations with additional consideration of the underlying structure. Altogether, the present study makes an important contribution to the reliable and valid assessment of the patient-GP interaction with regard to obesity counseling in primary care.

## Additional file

Additional file 1: **Table S1.** Factor loadings for PACIC (promax rotation) - four-factor model. **Table S2.** Factor loadings for 5A (promax rotation) - four-factor model. (DOCX 20 kb)

## Abbreviations

BL: Baseline Assessment
BMI: Body Mass Index
CCM: Chronic Care Model
CFA: Confirmatory Factor Analysis
CFI: Comparative Fit Index
EFA: Exploratory Factor Analysis
FU: Follow-Up
GP: General Practitioners
ICC: Intraclass Correlation Coefficient
KMO: Kaiser-Meyer-Olkin-Criterion
PA: Parallel Analysis
PACIC: Patient Assessment of Chronic Illness Care
PCA: Principal Component Analysis
RMSEA: Root Mean Square Error of Approximation
SRMR: Standardized Root Mean Square Residuals
WHO: World Health Organization
