# Supplementary material for: Psychometric properties of the Patient Assessment of Chronic Illness Care measure (PACIC-5A) among patients with obesity
Source: BMC Health Serv Res. 2019 Jan 23;19:61. doi: 10.1186/s12913-019-3871-1 (PMC6343299; doi:10.1186/s12913-019-3871-1)
Supplement: Supplementary file 1 — Table S1. Factor loadings for PACIC (promax rotation) - four-factor model. Table S2. Factor loadings for 5A (promax rotation) - four-factor model. (DOCX 20 kb) [file 12913_2019_3871_MOESM1_ESM.docx]

**Additional file**

Table S1 Factor loadings for PACIC (promax rotation) – four-factor model

| Items | Factors | | | |
| --- | --- | --- | --- | --- |
|  | 1 | 2 | 3 | 4 |
| 1 | ,785 | ,056 | -,047 | ,058 |
| 2 | ,699 | ,106 | -,144 | ,161 |
| 3 | ,250 | ,083 | ,523 | -,190 |
| 4 | ,114 | ,703 | -,254 | ,160 |
| 5 | ,806 | -,101 | ,074 | -,095 |
| 6 | ,827 | -,064 | -,114 | -,017 |
| 7 | ,886 | -,050 | ,017 | -,033 |
| 8 | ,782 | ,001 | -,007 | ,052 |
| 9 | -,098 | ,912 | -,180 | ,187 |
| 10 | ,608 | -,217 | -,076 | ,270 |
| 11 | ,046 | ,499 | ,279 | -,216 |
| 12 | ,623 | ,017 | ,349 | -,025 |
| 13 | ,229 | ,479 | ,134 | ,186 |
| 14 | ,475 | ,319 | ,174 | ,044 |
| 15 | ,579 | ,246 | ,146 | -,045 |
| 16 | -,159 | ,762 | ,090 | -,293 |
| 17 | ,280 | -,143 | ,052 | ,662 |
| 18 | -,047 | ,090 | ,171 | ,835 |
| 19 | -,319 | ,102 | ,798 | ,409 |
| 20 | ,028 | -,252 | ,954 | ,123 |

Table S2 Factor loadings for 5A (promax rotation) – four-factor model

| Items | Factors | | | |
| --- | --- | --- | --- | --- |
|  | 1 | 2 | 3 | 4 |
| 1 | ,818 | ,067 | ,070 | -,134 |
| 2 | ,798 | -,021 | ,165 | -,164 |
| 3 | ,028 | ,434 | -,028 | ,338 |
| 4 | ,178 | ,113 | ,656 | -,166 |
| 6 | ,783 | -,092 | ,111 | -,157 |
| 7 | ,872 | ,120 | -,072 | -,113 |
| 8 | ,832 | ,038 | ,021 | -,095 |
| 9 | -,055 | ,315 | ,766 | -,087 |
| 10 | ,826 | -,235 | -,088 | -,098 |
| 11 | -,096 | ,665 | ,118 | ,072 |
| 12 | ,519 | ,280 | ,000 | ,222 |
| 13 | ,276 | ,286 | ,370 | ,112 |
| 14 | ,438 | ,404 | ,178 | ,042 |
| 15 | ,511 | ,421 | ,084 | -,025 |
| 16 | -,361 | ,699 | ,370 | -,060 |
| 17 | ,554 | -,363 | ,115 | ,276 |
| 18 | ,281 | -,451 | ,419 | ,543 |
| 19 | -,303 | ,111 | ,139 | ,945 |
| 20 | -,083 | ,251 | -,265 | ,871 |
| 21 | ,491 | ,159 | -,043 | ,355 |
| 22 | ,379 | ,599 | -,167 | -,012 |
| 23 | ,095 | ,656 | ,147 | ,085 |
| 24 | ,715 | ,086 | -,223 | ,225 |
| 25 | ,701 | ,131 | -,034 | ,088 |
| 26 | -,099 | ,112 | ,658 | ,219 |
